# Electroacupuncture pretreatment alleviates myocardial ischemia injury via brown adipose tissue and BMP3b/Smad1/5 pathway in mice

**DOI:** 10.1080/21623945.2025.2586870

**Published:** 2025-11-09

**Authors:** Yuhang Yan, Danying Qian, Xiaohan Lu, Xiaoer Liu, Meiling Yu, Shengfeng Lu

**Affiliations:** a Key Laboratory of Acupuncture and Medicine Combination of Ministry of Education, Nanjing University of Chinese Medicine, Nanjing, China; b Department of Massage, The First People’s Hospital of Xiaoshan District/Xiaoshan Affiliated Hospital of Wenzhou Medical University, Hangzhou, China; c School of Elderly Care Services and Management/School of Aging Industry, Nanjing University of Chinese Medicine, Nanjing, China; d Jiangsu Province Engineering Research Center of TCM Intelligence Health Service, Nanjing University of Chinese Medicine, Nanjing, China

**Keywords:** Electroacupuncture, acute myocardial infarction, brown adipose tissue, bone morphogenetic protein 3b, Smad1/5

## Abstract

Acute myocardial infarction (AMI) is a critical condition that induces myocardial ischaemic injury and necrosis, requiring timely intervention to improve outcomes. Emerging evidence suggests that brown adipose tissue (BAT) plays a crucial role in cardiac protection by releasing bone morphogenetic protein 3b (BMP3b), which targets the Smad1/5 pathway in the heart. Here, we investigated whether Electroacupuncture (EA) pretreatment alleviates myocardial injury by activating BAT in AMI mice. The AMI model was established by left coronary artery ligation in male C57BL/6J mice, and EA was applied before AMI model establishment. Comprehensive assessments included morphological and molecular analyses of BAT, cardiac function evaluation, and infarct area measurements. The BMP3b/Smad1/5 signalling pathway was detected in BAT and the heart. Finally, we used mice with scapular BAT removed to verify the pivotal role of BAT in reducing myocardial injury caused by EA. The results showed that EA at ST25 enhanced BAT activation, upregulated BMP3b expression, promoted Smad1/5 phosphorylation, and increased the anti-apoptotic factor Bcl-xL, reducing myocardial damage. However, the protective effect of EA was not observed in the BAT-deficient mice. These findings suggest that EA at ST25 is a promising approach to reduce myocardial injury via the BAT and BMP3b/Smad1/5 pathways.

## 1. Introduction

Acute myocardial infarction (AMI) is a leading cause of death worldwide. It is occurring at younger ages due to factors such as sedentary lifestyle, substance misuse, and psychosocial stress [1,2]. Coronary artery occlusion leads to continuous ischaemia and hypoxia in cardiomyocytes, which triggers a series of inflammatory reactions, apoptosis, and necrosis of cardiomyocytes, eventually leading to heart failure [3]. Therefore, effective prevention and early intervention are essential for reducing AMI-related morbidity and mortality.

It has previously been found that brown adipose tissue (BAT) is activated after myocardial infarction, which is present in the interscapular region in rodents and the subclavicular region in humans [4]. BAT is an important thermogenic tissue and also plays an important role in regulating systemic and cardiac metabolism in mice and humans [5,6]. It is activated by norepinephrine (NE) released from the sympathetic nervous system. NE binds to β3-adrenergic receptors (β3-AR) in brown adipocytes, promoting uncoupling protein 1 (UCP1) expression and thermogenesis [7,8]. Recent studies have shown that increasing the mass of BAT in mice enhances the tolerance of the myocardium to ischaemia-reperfusion injury, thereby improving the prognosis of AMI and heart failure [9]. Moreover, its cardioprotective effect is produced by the secretion of the myocardial protective factor Bone morphogenetic protein 3b (BMP3b), which binds to BMP receptors in the heart and promotes the phosphorylation of downstream signalling pathways Smad1/5, thereby protecting cardiomyocytes from cell damage and oxidative stress caused by long-term hypoxia [10,11]. Therefore, BAT and BMP3b/Smad1/5 signalling pathways may be key targets for the treatment of AMI.

Electroacupuncture (EA) is a minimally invasive external therapy of traditional Chinese medicine that involves insertion into the subcutaneous muscles, fascia, and other tissues to exert effects on corresponding tissues, organs, or the whole body through mechanical and electrical stimulation. Previous studies have confirmed that EA has a specific regulatory effect on adipose tissue [12,13]. Notably, the Tianshu acupoint (ST25), located in the abdomen, can excite the sympathetic nerve on BAT and promote browning of white adipose tissue (WAT), thereby increasing heat production and reducing the weight of diabetic model rats [14]. Moreover, research has shown that abdominal EA pre-treatment can induce cardioprotection by activating the sympathetic nervous system [15]. However, whether EA at ST25 has cardioprotective effects and whether its mechanism is related to BAT has not been fully investigated. Therefore, we hypothesized that EA pre-treatment at ST25 might exert cardioprotective effects through BAT. In this study, we ligated the left anterior descending coronary artery of mice to simulate myocardial infarction in patients, considering the possibility of BAT activation in AMI injury and the role of BAT and the BMP3b/Smad1/5 signalling pathway in alleviating myocardial injury by EA.

## 2. Materials and methods

### 2.1. Animals and study design

Healthy eight-week-old male C57BL/6J mice (22 ± 2 g) were purchased from Huangchuang Sino Co., Ltd. (Certificate No. SCXK 2020–0009, Jiangsu, China) and were housed under standard conditions at a constant temperature (24 ± 2 °C) and a 12/12 h light-dark cycle. Food and water were provided ad libitum. After one week of environmental adaptation, the mice were divided into 6 groups using a random number table: sham-operated (Sham), MI, EA + MI (EA), BATectomy + Sham, BATectomy + MI, and BATectomy + ST25 EA + MI (BATectomy + EA) groups. Due to the high mortality rate of surgical modeling, a total of 66 mice were prepared for molecular and morphological experiments according to our previous experimental experience. Among them, the number of mice in the sham group was 9, and the number of mice in the other experimental groups was 12. During the experiment, 10 rats died, and 2 rats failed in modeling (see 2.2 for the success criteria). These mice were excluded, and the rest were used for experiments. The mortality rates in each group are shown in Table 1. Cardiac function was assessed using electrocardiography (ECG) and echocardiography 24 h after modeling. The mice were then anesthetized and killed by cervical dislocation. Plasma, heart, and BAT samples were collected for enzyme-linked immunosorbent assay (ELISA), triphenyltetrazolium chloride (TTC) staining, haematoxylin-eosin (HE) staining, quantitative real-time polymerase chain reaction (RT-qPCR), and western blotting (WB). In order to make the experimental data more objective, we used the working mode of the assembly line. Xiaoer Liu carried out the experimental grouping, Yuhang Yan carried out the intervention, Danying Qian carried out the modelling, Xiaohan Lu carried out the ECG and echocardiography detection, Yuhang Yan carried out the morphological and molecular detection, and the final data analysis. During the experiment, the mice in each group were randomly selected by an assistant so that the experimenter would not be aware of the grouping. All experiments were approved by the Experimental Animal Welfare Ethics Committee of Nanjing University of Chinese Medicine (Approval No. 202402A032). The study was designed and reported following the ARRIVE guidelines.

**Table 1.** Evaluation of animal survival rate.

Group	Number	Death	Failure of modelling	Survival rate (%)
Sham	9	0	0	100
MI	12	2	1	75
EA	12	3	0	75
BATectomy + Sham	9	0	0	100
BATectomy + MI	12	3	0	75
BATectomy + EA	12	2	1	75

### 2.2. Model establishment

Left anterior descending coronary artery (LAD) ligation is a commonly used animal model of AMI [16]. Mice were anesthetized with 5% isoflurane with high-purity oxygen, maintained under anaesthesia with 1–2% isoflurane, and fixed on a heating pad at (37 ± 1℃). The mouse heart was extruded from the 3rd and 4th left intercostal spaces of the thoracic cavity. The LAD was permanently ligated using a 6–0 Nylon thread to induce myocardial infarction. The criteria for successful modelling were upward elevation of the arch of the ST segment of lead II of the limb [16]. After surgery, the mice were placed on a heating pad until they were awake. Mice in the Sham group were subjected to the same procedure, except for the LAD ligation.

### 2.3. Electroacupuncture intervention

EA was conducted with pairs of unipolar stainless-steel acupuncture needles (0.16 × 7 mm, Beijing Zhongyan Taihe Medical Instrument Co., Ltd, Jiangsu, China). The electrical current range was set at 1.0 mA, and the stimulation lasted for 20 min, with a frequency of 2/15 Hz, controlled using an EA apparatus (HANS-200A, Han Acuten, Beijing, China). Two pairs of acupuncture needles were perpendicularly inserted into the bilateral ST25 (located 5 mm lateral to the intersection point of the upper 2/3 and lower 1/3 on the line between the xiphoid process and upper margin of the pubic symphysis) at a depth of 3–5 mm [17]. Myocardial infarction modelling was performed within 10–15 min after EA.

### 2.4. Surgical removal of BAT

Before EA pretreatment, the BAT of the mice in the BATectomy group was surgically removed [18]. The mice were anesthetized, and the shoulder blades were shaved. A 1–1.5 cm incision was made vertically with surgical scissors, exposing the fat depot on the shoulder blades of the mice. The two leaves of the dark triangle BAT were removed intact with surgical scissors, and the skin was sutured. During and after surgery, a heat pad was used for warming until consciousness was fully recovered.

### 2.5. Measurement of electrocardiogram recording

Electrocardiography was performed 24 h after the operation. Mice were anesthetized with isoflurane (1.5%). Three metal electrodes were inserted under the skin of the right wrist and mouse ankles. A surface lead II electrocardiogram (ECG) was obtained. The subsequent 1-minute recording of the ECG was analysed using Lab Chart 8.2.3 (ADInstruments, Australia).

### 2.6. Measurement of echocardiography

All mice underwent transthoracic echocardiography under 1–1.5% isoflurane anaesthesia. The anterior and posterior left ventricular walls were observed with M-mode maps, and the left ventricular end-diastolic diameter (LVEDD) and left ventricular end-systolic diameter (LVESD) were measured. Left ventricular ejection fraction (EF = (EDV-ES)/EDV × 100%) and fractional shortening (FS = (LVEDD−LVESD)/LVEDD × 100%) were calculated using the Teichholz correction formula [19].

### 2.7. Triphenyltetrazolium chloride staining

In each group, four mice were selected and deeply anesthetized with 5% isoflurane, followed by cervical dislocation to sacrifice and remove their hearts. The hearts were placed in a freezer at −20°C for 1 h. Subsequently, the regions below the ligation lines of the hearts were sliced into five sections using a mouse heart slicing mould. The Hearts were stained with 1.5% TTC solution at 37°C for 15 min and immersed in paraformaldehyde for 8 - 10 min before being photographed. The infarct area of mice in each group was calculated using the ImageJ software (National Institutes of Health, Germany).

### 2.8. Haematoxylin staining

BAT was removed from the shoulders of the mice and fixed in 4% paraformaldehyde for 24 h. Paraffin embedding was performed after dehydration using an ethanol solution. Cut the wax block 4 μm. The sections were dewaxed with xylene, rehydrated with an alcohol solution, and then stained with haematoxylin for 2 min. Slices were rinsed with running water and faded in a 1% hydrochloric acid ethanol solution. After rinsing, the ethanol was dehydrated and stained with a 0.5% eosin ethanol solution. The ethanol was dehydrated and placed in a transparent, neutral xylene gum seal. The images were obtained using an automatic quantitative pathological imaging system (Vectra 3.0, PerkinElmer, America), and ImageJ software was used for computational analysis.

### 2.9. Enzyme-linked immunosorbent assay

Blood samples (0.75 ml) were collected from the posterior orbital venous plexus of the mice. After standing for 2 h, the samples were centrifuged, and the supernatant was extracted. The experiment was performed in accordance with the manufacturer’s instructions of the kit (JM‑11532M1, LA128163H, Nanjing Jinyibai Biotechnology Co. Ltd., Jiangsu, China). The absorbance values were read at a wavelength of 450 nm using an enzyme-labelled instrument, and a standard curve was plotted to calculate the contents of cardiac troponin T (cTnT) and BMP3b in the serum of each mouse.

### 2.10. Quantitative real-time polymerase chain reaction

The expression levels of β3-adrenergic receptor (β3-AR), uncoupling protein 1 (UCP1), and BMP3b mRNA in the BAT of mice were determined by RT-qPCR. BAT was collected from the scapular region, and a total RNA extraction solution (RC101-01, Vazyme, Jiangsu, China) was added. The tissue was ground in a ball mill, and RNA was extracted from BAT. After measuring concentration and purity, reverse transcription was performed to synthesize stable cDNA. Primer sequences for β3-AR, UCP1, BMP3b, and the internal reference GAPDH were designed (primer sequences are shown in Table 2). According to the steps described in the SYBR kit (Q311-02, Vazyme, Jiangsu, China) manual, the primer and stable cDNA mixture were added to 384-well plates with three replicates for each sample. Amplification was performed by PCR. The reaction conditions were as follows: pre-denaturation at 95°C for 30 s, one cycle; denaturation at 95°C for 10 s, annealing at 60°C for 30 s, 40 cycles. CT values of the samples were obtained. Using GAPDH as an internal reference, the relative expression levels of the target genes were calculated based on 2*(− ΔΔCt).

**Table 2.** Sequence of genes.

Gene Name	Forward	Reverse
UCP1	CACCTTCCCGCTGGACACT	CCCTAGGACACCTTTATACCTAATGG
β3-AR	AGGTCAATGAAGGGGTCGTT	AACGCAAAGGGTTGGTGACA
BMP3b	CTATCCACATGCTCAGGCTCT	GCTTTTGGTCGATCATTTCCAGC
GAPDH	AAATGGTGAAGGTGAAGGTCGGTGTG	AGGTCAATGAAGGGGTCGTT

### 2.11. Western blotting

WB was used to detect the expression levels of B-cell lymphoma-extra large (Bcl-xL), p-Smad1/5, Smad1, and BMP3b in the myocardial tissue of the infarcted area of mice in each group, as well as the expression levels of UCP1 and BMP3b in BAT. RIPA cell lysis buffer (P0013B, Beyotime, Shanghai, China) and protease inhibitors (P1045, Beyotime, Shanghai, China) were added, and the tissues were ground using a ball mill. Protein concentration was determined using the BCA method (23227, Thermo Fisher, USA). Heating ensures the complete denaturation of proteins. 10% SDS-PAGE was employed. Proteins (100 μg) were added to each loading well and transferred to a PVDF membrane. After blocking with a rapid blocking solution for 30 min, primary antibodies (Bcl-xL, T40057, Abmart, China, 1:1000; p-Smad1/5, 9516s, Cell Signaling Technology, USA, 1:1000; Smad1, 10,429–1-AP, Proteintech, China, 1:5000; UCP1, ab10983, Abcam, UK,; 1:1500; BMP3b, DF9282, Affinity, Melbourne, 1:1000; GAPDH, 60,004–1-LG, Proteintech, China, 1:5000) were added and incubated at 4°C overnight. The following day, the secondary antibody (SA00001-1, Proteintech, China, 1:5000; SA00001-2, Proteintech, China, 1:5000) was incubated at room temperature for 1 h. The protein bands were developed using an ECL luminescence solution (P0018AS, Beyotime, Shanghai, China), and the grey values of the bands were calculated using the ImageJ image analysis software. The ratio of the grey value of the target protein band to that of the internal reference protein band was taken as the relative expression level of the target protein.

### 2.12. Statistical analysis

All statistical analyses were performed using the SPSS software (version 29.0; IBM, USA). The normality of the data was checked using the Shapiro-Wilk test. Homogeneity of variance was assessed using Levene’s test. The measurement data conforming to normal distribution were analysed using the unpaired t-test, and the measurement data not conforming to normal distribution were analysed using the Mann-Whitney rank sum test. One-way analysis of variance (ANOVA) was used to compare the groups. All data are presented as the mean ± SEM. Statistical significance was set at *p* < 0.05.

## 3. Results

### 3.1. ST25 EA pretreatment alleviates myocardial injury after AMI

**Figure 1.**
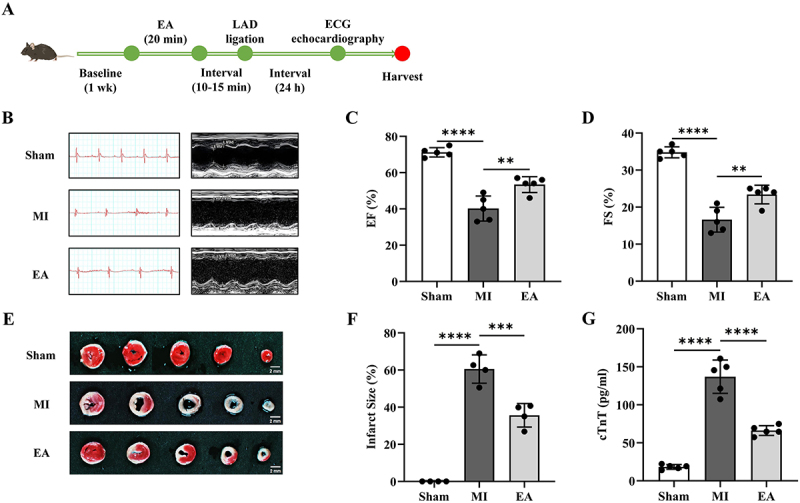
Cardioprotective effect of EA pretreatment at ST25 on AMI mice. (a) flowchart of animal experiment procedure. (b) Representative records of ECG ii leads and echocardiography in each group. (c) calculation and analysis of left ventricular ejection fraction in each group (*n* = 5). (d) calculation and analysis of left ventricular fractional shortening in each group (*n* = 5). (e) Representative images of TTC staining in the hearts of mice in each group. (f) calculation and analysis of cardiac infarction size of mice in each group (*n* = 4). (g) comparison of serum cTnT levels in each group (*n* = 5). Data are expressed as the mean ± standard error of the mean. **p < .01, ***p < .001，****p < .0001. EA: electroacupuncture; LAD: left anterior descending coronary artery; ECG: electrocardiogram; Sham: the sham operation group; MI: acute myocardial infarction model group; EF: ejection fraction; FS: fraction shortening; cTnT: cardiac troponin T.

First, we explored whether ST25 protected against myocardial damage. ECG, echocardiography, serum cTnT, and TTC staining were used to evaluate the cardiac function of AMI mice 24 h after cardiac ligation (Figure 1(A)). We observed that compared with the sham group, the ECG II lead ST-segment was elevated, the Q-wave was depressed, and the left ventricular anterior wall motion was weakened in the MI group. Compared to the MI group, the ST segment of the ECG in the EA group was decreased, the Q-wave amplitude was decreased, and the left ventricular anterior wall motion was enhanced (Figure 1(B)). Calculation and analysis of left ventricular EF and FS showed that left ventricular function decreased in the MI group, whereas EF and FS increased in the EA group (Figure 1(C-D)). We then measured the myocardial infarction size and serum cTnT content in each group of mice. It was found that the infarct size and cTnT level were significantly reduced in the EA group compared with the MI group (Figure 1(E-G)). These results indicate that EA pre-treatment at ST25 can alleviate myocardial injury and improve cardiac function.

### 3.2. ST25 EA pretreatment enhances the activation of bat and the secretion of BMP3b after AMI

**Figure 2.**
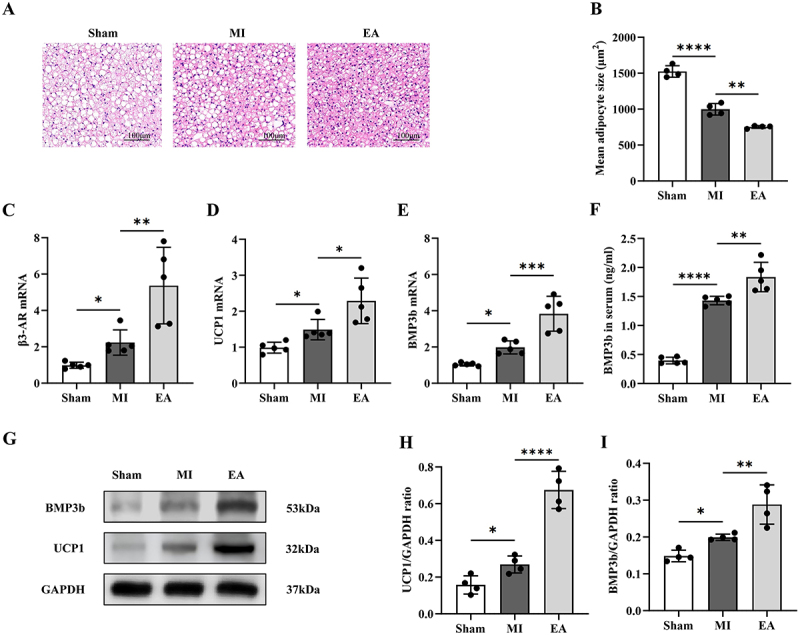
Effect of ST25 EA pretreatment on bat pathology and the expression of BMP3b in AMI mice. (a) bat he staining represented pictures of mice in each group (20×). (b) the average area of adipocytes in bat of mice in each group was calculated according to Figure a (*n* = 4). (c) comparison of the relative expression levels of β3-AR mRNA in bat of mice in each group (*n* = 5). (d) comparison of the relative expression levels of UCP1 mRNA in bat of mice in each group (*n* = 5). (e) comparison of the relative expression levels of BMP3b mRNA in bat of mice in each group (*n* = 5). (f) comparison of serum BMP3b levels among all groups (*n* = 5). (g) wb bands of UCP1 and BMP3b in bat of mice in each group represented pictures. (h) comparison of the relative expression of UCP1 protein in bat of mice in each group (*n* = 4). (i) comparison of the relative expression of BMP3b protein in bat of mice in each group (*n* = 4). Data are expressed as the mean ± standard error of the mean. **p < .05, ***p < .01, ****p < .001, *p < .0001. Sham: the sham operation group; MI: acute myocardial infarction model group; EA: electroacupuncture; β3-AR: β3-adrenergic receptor; UCP1: uncoupling protein 1; BMP3b: bone morphogenetic protein 3b.

This is because of the effect of EA on BAT and its protective effect on the heart [10,14]. We verified the effect of EA at ST25 on pathological changes and BMP3b-related pathways in BAT of AMI mice. The volume of brown adipocytes in the MI group was lower than that in the Sham group. The brown adipocytes in the EA group were smaller and had more nuclei than those in the MI group (Figure 2(A-B)). The results showed that EA promoted BAT activation in AMI mice. We then detected the secretion of BMP3b and the expression of related substances in mouse BAT. The results showed that BMP3b’s upstream signals β3-AR and UCP1 were activated in myocardial infarction, and this change was enhanced by EA at ST25 (Figure 2(C-D, G-H)). Additionally, the increased expression of BMP3b in BAT and serum of the EA group represents the migration of BMP3b, which further proves that EA at ST25 enhances the secretion of BMP3b in BAT of AMI mice (Figure 2E-G, I).

### 3.3. ST25 EA pretreatment reduces cardiomyocyte apoptosis via BMP3b/Smad1/5

**Figure 3.**
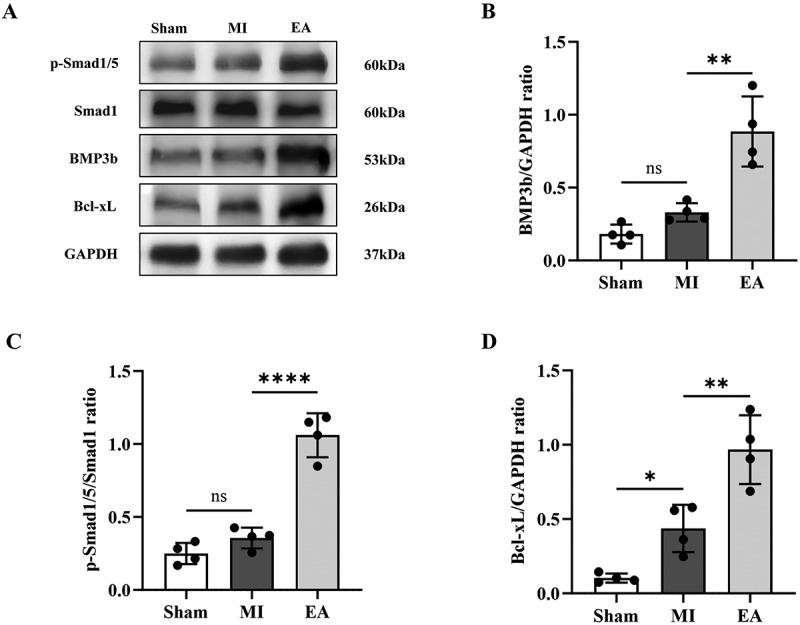
Effect of ST25 EA pretreatment on the expression of BMP3b/Smad1/5 signalling pathway in the heart of AMI mice. (a) the wb bands of p-Smad1/5, Smad1, BMP3b, and Bcl-xL in the myocardial tissue of mice in each group represented pictures. (b) comparison of the relative expression of BMP3b protein in myocardial tissue of mice in each group (*n* = 4). (c) comparison of the relative expression of p-Smad1/5 protein in myocardial tissue of mice in each group (ratio of p-Smad1/5 to Smad1) (*n* = 4). (d) comparison of the relative expression of Bcl-xL protein in myocardial tissue of mice in each group (*n* = 4). Data are expressed as the mean ± standard error of the mean. **p < .05，***p < .01，****p < .0001. ns: not significant; Sham: the sham operation group; MI: acute myocardial infarction model group; EA: electroacupuncture; BMP3b: bone morphogenetic protein 3b; Bcl-xL: B-cell lymphoma-extra large.

Next, we examined whether the myocardial protection of EA at ST25 was related to the BMP3b/Smad1/5 signalling pathway. The results showed that the content of BMP3b and phosphorylation of Smad1/5 in the hearts of mice increased after myocardial infarction, but the difference was not statistically significant. After EA, the content of BMP3b and phosphorylation of Smad1/5 in the heart were significantly increased (Figure 3A-C). The downstream signal Bcl-xL in the BMP3b/Smad1/5 pathway was also examined, and a consistent trend was observed (Figure 3A, D). These results indicate that EA at ST25 can reduce myocardial injury by enhancing the anti-apoptotic effects of the myocardium.

### 3.4. The cardioprotective effect of ST25 EA pretreatment vanishes after bat removal

**Figure 4.**
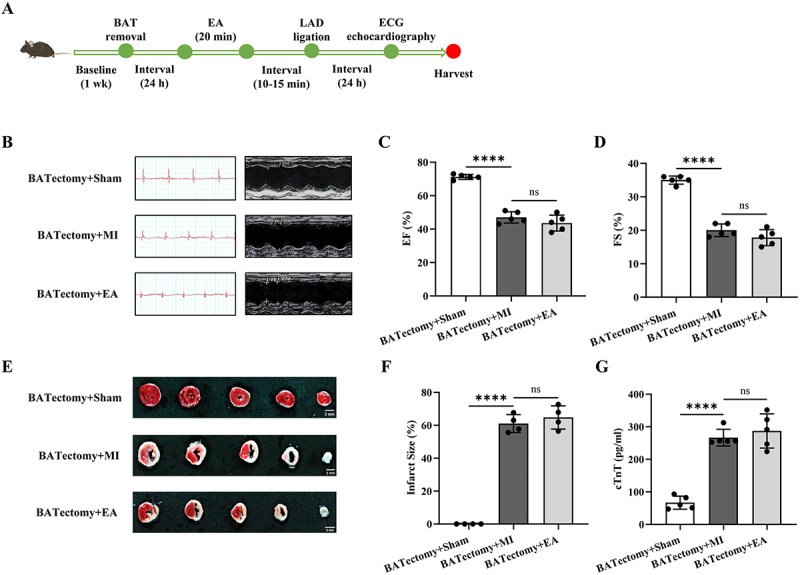
Cardioprotective effect of ST25 EA pretreatment on AMI mice after bat removal. (a) flowchart of BATectomy animal experiment procedure. (b) Representative records of ECG ii leads and echocardiography in each BATectomy group. (c) calculation and analysis of left ventricular ejection fraction in each BATectomy group (*n* = 5). (d) calculation and analysis of left ventricular fractional shortening in each BATectomy group (*n* = 5). (e) Representative images of cardiac TTC staining in each group of BATectomy mice. (f) calculation and analysis of cardiac infarction size of mice in each BATectomy group (*n* = 4). (g) comparison of serum cTnT levels in each BATectomy group (*n* = 5). Data are expressed as the mean ± standard error of the mean. **p < .0001. ns: not significant; BAT: brown adipose tissue; EA: electroacupuncture; LAD: left anterior descending coronary artery; ECG: electrocardiogram; BATectomy+Sham: the bat removal + sham operation group; BATectomy+MI: the bat removal + acute myocardial infarction model group; BATectomy+EA: the bat removal + electroacupuncture group; EF: ejection fraction; FS: fraction shorting; cTnT: cardiac troponin T.

To test the necessity of BAT for cardioprotection of EA at ST25, we excised BAT in the scapular region of mice before EA. The cardiac function of the mice 24 h after infarction was evaluated (Figure 4(A)). First, compared with the groups without BAT removal, BAT removal had no significant effect on the cardiac function of the mice (Figures 1B and 4B). The results showed that ST25 EA had no effect on the pathological changes in ECG and cardiac function in AMI mice. There was even a tendency for the damage to worsen (Figure 4(B-D)). The myocardial protective effect of ST25 was also lost in terms of myocardial infarction size and serum cTnT results (Figure 4(E-G)).

### 3.5. The anti-apoptotic pathway of ST25 disappears after bat removal

**Figure 5.**
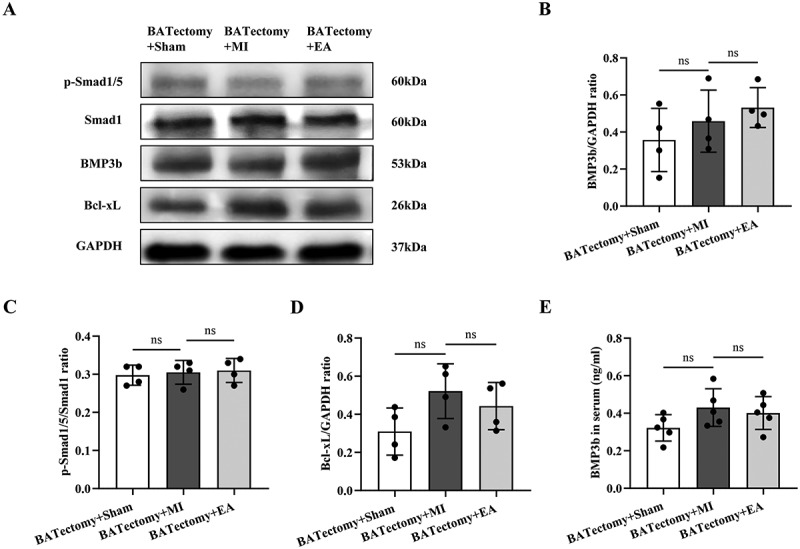
Effects of ST25 EA pretreatment on the expression of BMP3b/Smad1/5 signalling pathway in AMI mice after bat removal. (a) wb bands representing p-Smad1/5, Smad1, BMP3b, and Bcl-xL in the myocardial tissue of mice in each BATectomy group. (b) comparison of the relative expression of BMP3b protein in myocardial tissue of mice in each BATectomy group (*n* = 4). (c) comparison of the relative expression of p-Smad1/5 protein in myocardial tissue of mice in each BATectomy group (ratio of p-Smad1/5 to Smad1) (*n* = 4). (d) comparison of the relative expression of Bcl-xL protein in myocardial tissue of mice in each BATectomy group (*n* = 4). (e) comparison of serum BMP3b levels among all BATectomy groups (*n* = 5). Data are expressed as the mean ± standard error of the mean. ns: not significant; BATectomy+Sham: the bat removal + sham operation group; BATectomy+MI: the bat removal + acute myocardial infarction model group; BATectomy+EA: the bat removal + electroacupuncture group; BMP3b: bone morphogenetic protein 3b; Bcl-xL: B-cell lymphoma-extra large.

Next, we examined changes in the BMP3b/Smad1/5 signalling pathway in the hearts of each group of mice after BAT removal. The results showed that there were no significant differences in the BMP3b content and the degree of phosphorylation of Smad1/5 in myocardial tissue among all groups (Figure 5(A-C)). This indicates that regulation of the BMP3b/Smad1/5 signalling pathway by ST25 is dependent on BAT. Accordingly, the upregulation effect of ST25 EA on the anti-apoptotic protein Bcl-xL was abolished (Figure 5(D)). Serum levels of BMP3b did not increase in either the MI or EA groups (Figure 5(E)). These results indicated that BAT is necessary for the protective effects of EA at ST25.

## 4. Discussion

**Figure 6.**
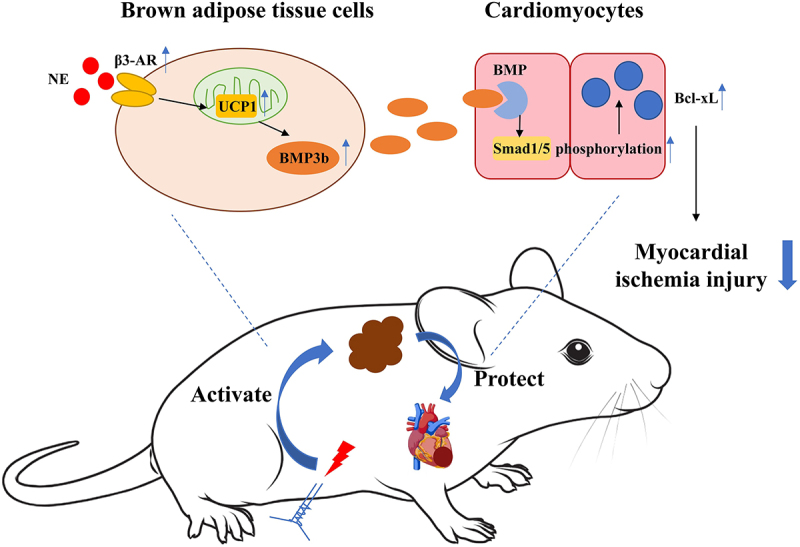
Schematic diagram of the effect of EA on reducing myocardial ischaemic injury through the BMP3b/Smad1/5 pathway in bat. NE: norepinephrine; β3-AR: β3-adrenergic receptor; UCP1: uncoupling protein 1; BMP3b: bone morphogenetic protein 3b; BMP: bone morphogenetic protein receptors; Bcl-xL: B-cell lymphoma-extra large.

This study primarily investigated the myocardial protective effects of EA at ST25, focusing on its ability to activate BAT and its potential link to the BMP3b/Smad1/5 signalling pathway. Our findings show that EA pretreatment at ST25 enhances the activation of BAT and the BMP3b/Smad1/5 signalling pathway, upregulating its upstream signals, such as β3-AR and UCP1, and its downstream anti-apoptotic factor Bcl-xL, ultimately alleviating myocardial injury (Figure 6). This effect vanishes after the BAT is removed. These results are consistent with previous reports that BAT ablation can aggravate pathological remodelling of the heart [20]. Detailed introductions are as follows:

Research has indicated that abdominal EA pretreatment demonstrates a strong myocardial protective effect (reducing the myocardial infarction area) [15]. Therefore, we used TTC staining, ECG, echocardiography, and serum cTnT levels to evaluate the cardioprotective effects of EA. Our results show that after 4 h of AMI modelling, the ST segment of the electrocardiogram in both the MI and EA groups was elevated, with an amplitude greater than 0.1 mV, indicating the success of the model establishment. At 24 h post-modelling, our results revealed that the infarction area in the EA group was smaller than that in the MI group, and its cardiac function was superior to that of the MI group. These findings prove that ST25 EA pre-treatment has a protective effect against myocardial ischaemic injury. This may provide a potential approach to the prevention and treatment of AMI.

Apoptosis of myocardial cells, driven by continuous ischaemia and hypoxia, is a key contributor to myocardial injury after AMI [21]. The SMAD family plays a crucial role in regulating myocardial apoptosis, fibrosis, and ventricular remodelling following myocardial infarction [22]. Among them, the phosphorylation of Smad1/5 is closely associated with the apoptosis and oxidative stress of cardiomyocytes induced by ischaemia and hypoxia [11,23]. However, the Smad1/5 cascade is primarily triggered by bone morphogenetic protein receptors (BMP), which mediate a myocardial protective effect by enhancing the expression of the anti-apoptotic protein Bcl-xL in the heart, thereby alleviating cardiomyocyte apoptosis [24]. BMP receptors, which belong to the TGF-β superfamily (including TGF-β and BMP), are activated following myocardial infarction and play a significant role in regulating fibroblast phenotype and cardiac fibrosis through Smad-mediated signalling [25]. Recent studies suggest that BMP3b, a protein secreted by BAT, binds to BMP receptors and protects the heart by promoting the phosphorylation of Smad1/5 [10]. A deficiency of BMP3b results in left ventricular hypertrophy and decreased myocardial contractility [26]. Previous studies have demonstrated that EA can reduce myocardial ischaemia-reperfusion injury by mitigating myocardial cell apoptosis [27]. In our study, compared with the model group, ST25 EA pretreatment led to increased expression of the anti-apoptotic factor Bcl-xL, elevated BMP3b levels, and increased phosphorylation of Smad1/5 in the heart. Furthermore, serum BMP3b levels were elevated, suggesting the migration of BMP3b from BAT to the heart, where it interacts with BMP receptors to influence downstream Smad1/5 signalling. These results suggest that EA at ST25 reduces myocardial ischaemic injury by enhancing the anti-apoptotic effect of the myocardium, potentially through the BMP3b/Smad1/5 pathway.

BAT is activated in response to cold exposure or long-term exercise [28,29]. Activation of the sympathetic nerves surrounding BAT leads to the release of NE, which binds to β3-AR in BAT. This increases the expression of UCP1 in BAT, promoting browning of WAT and increasing BAT mass [30,31]. BAT plays a significant role in various metabolic and endocrine disorders [31,32]. UCP1-dependent activation of BAT is capable of accelerating cardiometabolic remodelling and reducing the initial hypertrophic and fibrotic response to pathological stress [33]. Moreover, recent research indicates that increasing the mass of BAT in mice can enhance the heart’s tolerance to ischaemia-reperfusion injury and improve the prognosis of AMI and concomitant heart failure [4,9]. Conversely, the absence of BAT can undermine the body’s resistance to cardiovascular diseases such as acute myocardial infarction induced by long-term exercise [34]. It is well known that ST25 is a commonly used acupuncture point in traditional Chinese medicine for weight loss and gastrointestinal dysfunction [35–37]. Recent findings have suggested that ST25 EA is closely associated with BAT activation. For example, EA at ST25 has been shown to enhance heat production in obese diabetic mice, reduce body weight, and lower blood glucose levels by stimulating sympathetic nerve activity in BAT and increasing the expression of UCP1 [12,14]. The specific mechanisms are as follows. Activated sympathetic nerves release NE, which binds to β3-AR. This binding activates adenylate cyclase, leading to the production of cyclic adenosine monophosphate (cAMP). The cAMP then activates protein kinase A (PKA), which in turn phosphorylates downstream targets such as p38 mitogen-activated protein kinase and the cAMP-response element binding protein (CREB). This ultimately upregulates the expression of thermogenic genes. This pathway is known as the NE-β3-AR-CREB-UCP1 signalling cascade [8]. UCP1 plays a critical role in activating BMP3b, which is closely associated with improved outcomes in myocardial infarction [9,10]. Deletion of UCP1 exacerbates myocardial injury during ischaemia-reperfusion [38]. Therefore, we examined pathological sections of BAT in AMI mice and the expression of β3-AR, UCP1, and BMP3b in BAT. Our results demonstrate that EA pretreatment at ST25 augmented the activation of BAT cells. Furthermore, EA at ST25 elevated the expression of β3-AR, UCP1, and BMP3b in AMI mice, and its efficacy was stronger than that in the MI group. This indicated that ST25 EA exerted myocardial protective effects by activating BAT and secreting BMP3b. To further confirm BAT’s role in the cardioprotective effect of ST25 EA, we performed BAT removal surgery. Our results showed that the myocardial protective effect of ST25 disappeared after BAT removal. In parallel, activation of the BMP3b/Smad1/5 signalling pathway and augmentation of the anti-apoptotic protein Bcl-xL were lost, indicating that BAT is essential for the myocardial protective effect of ST25 EA.

Given the activation of the sympathetic nervous system and BAT by EA at ST25, and the proliferation of BAT under exercise conditions [14,17,29]. We speculated that EA at ST25 May mimic the beneficial effects of exercise on cardiac function. This provides a novel perspective for the treatment of AMI and holds the potential to advance our understanding of the mechanisms of EA. However, this study has several limitations that warrant acknowledgement. the potential involvement of other BAT-derived secreted factors in alleviating myocardial injury through EA cannot be ruled out. Furthermore, the specificity of ST25, as well as its long-term effects on AMI-induced myocardial damage, remains unclear. These aspects represent important considerations for future research.

## 5. Conclusions

In conclusion, we demonstrated that ST25, a crucial acupoint responsible for treating gastrointestinal disorders, makes a novel contribution to the treatment of AMI. EA pretreatment at ST25 activated BAT, promoted BMP3b secretion, reduced myocardial apoptosis, and improved cardiac function via the BMP3b/Smad1/5 pathway. This novel non-pharmacological approach holds promise for AMI prevention and treatment.

### Ethics approval and consent to participate

The authors are accountable for all aspects of the work, ensuring that questions related to the accuracy or integrity of any part of the work are appropriately investigated and resolved. This study was approved by the Animal Ethics Committee of Nanjing University of Chinese Medicine Laboratory Animal Center and conducted in accordance with the National Institutes of Health’s Guide for the Care and Use of Laboratory Animals (Permit number: 202402A032).

## Data Availability

The data that support the findings of this study are available in figshare at https://doi.org/10.6084/m9.figshare.29587658.
